# Propensity score matching analysis comparing radical prostatectomy and radiotherapy with androgen deprivation therapy in locally advanced prostate cancer

**DOI:** 10.1038/s41598-022-16700-7

**Published:** 2022-07-21

**Authors:** Yu-Cheng Lu, Chao-Yuan Huang, Chia-Hsien Cheng, Kuo-How Huang, Yu-Chuan Lu, Po-Ming Chow, Yi-Kai Chang, Yeong-Shiau Pu, Chung-Hsin Chen, Shao-Lun Lu, Keng-Hsueh Lan, Fu-Shan Jaw, Pei-Ling Chen, Jian-Hua Hong

**Affiliations:** 1 Institute of Biomedical Engineering, National Taiwan University, No. 1, Changde St., Zhongzheng Dist., Taipei City, 10048 Taiwan; 2 Department of Urology, National Taiwan University Hospital, College of Medicine, National Taiwan University, Taipei, Taiwan; 3 Division of Radiation Oncology, Department of Oncology, National Taiwan University Hospital, College of Medicine, National Taiwan University, Taipei, Taiwan

**Keywords:** Urological cancer, Surgical oncology

## Abstract

To compare clinical outcomes between the use of robotic-assisted laparoscopic radical prostatectomy (RP) and radiotherapy (RT) with long-term androgen deprivation therapy (ADT) in locally advanced prostate cancer (PC), 315 patients with locally advanced PC (clinical T-stage 3/4) were considered for analysis retrospectively. Propensity score-matching at a 1:1 ratio was performed. The median follow-up period was 59.2 months (IQR 39.8–87.4). There were 117 (37.1%) patients in the RP group and 198 (62.9%) patients in the RT group. RT patients were older and had higher PSA at diagnosis, higher Gleason score grade group and more advanced T-stage (all *p* < 0.001). After propensity score-matching, there were 68 patients in each group. Among locally advanced PC patients, treatment with RP had a higher risk of biochemical recurrence compared to the RT group. In multivariate Cox regression analysis, treatment with RT plus ADT significantly decreased the risk of biochemical failure (HR 0.162, *p* < 0.001), but there was no significant difference in local recurrence, distant metastasis and overall survival (*p* = 0.470, *p* = 0.268 and *p* = 0.509, respectively). This information supported a clinical benefit in BCR control for patients undergoing RT plus long-term ADT compared to RP.

## Introduction

Prostate cancer (PC) is the second most frequent malignancy diagnosis made in men and the fifth leading cause of death worldwide^1^. There were over 1.4 million new cases of PC and 375,304 related deaths around the world in 2020^2^. Diagnostic estimates of loco-regional PC are over 90% in the United States^3^. In Taiwan, 58% of newly diagnosed cases of PC had loco-regional disease and, 11% had locally advanced disease between 2004 and 2012^4^. The management of locally advanced PC remains a challenge for urologists.

Traditionally, the risk of PC is stratified by serum prostate-specific antigen (PSA), Gleason score (GS) of the prostate biopsy and digital rectal exam (DRE). However, DRE is a subjective test with potential inter-observer variability and GS has shown discrepancy between prostate biopsy and radical prostatectomy specimens^5,6^. PSA values are also influenced by patient age and prostate volume. In the last decade, magnetic resonance imaging (MRI) of the prostate has become an essential diagnostic tool for local staging. The Prostate Imaging Reporting and Data System (PI-RADS) version 2 was updated in 2015 and developed to promote global standardization in the interpretation and reporting of the prostate MRI examination. Many studies have investigated the accuracy of MRI in local staging^7^.

Robotic-assisted laparoscopic radical prostatectomy (RP) with pelvic lymph node dissection and radiotherapy (RT) combined with long-term androgen deprivation therapy (LTADT) are currently standard treatment options for locally advanced PC^8^. Both treatments incorporate a multimodality approach in this high-risk population to improve oncologic outcomes at the expense of distinct potential complications.

RP has the advantage of more accurate disease staging with fewer bowel/rectal problems compared to RT, while anesthesia risk and associated higher risk of impotence and incontinence are still of concern^9^. On the other hand, RT with LTADT does not require hospitalization and has a lower risk of urinary incontinence but does convey a higher rate of radiation cystitis, bowel/rectal problems and possible side effects of ADT^9,10^.

Standard treatment options for locally advanced PC include RP with pelvic lymph node dissection or RT combined with hormone therapy, but the optimal therapy is still controversial. Several retrospective series found an advantage for RP but only on the basis of a low level of evidence, while others favored RT because of superior outcomes when adding LTADT^10^. However, RT dose and duration of ADT were heterogeneous. One ongoing prospective randomized study, SPCG-15 trial, comparing primary RP and RT plus ADT in locally advanced PC might provide valuable information in this specific population in the future^11^. To date, optimal management remains uncertain in locally advanced PC. In this study, we aimed to compare clinical outcomes in locally advanced PC between the use of RP and RT combined with LTADT.

## Methods

### Inclusion and exclusion criteria

Between January 1, 2008 and November 31, 2018, 533 PC patients with clinically T stage 3/4 defined by MRI were analyzed. There were 160 patients in the RP group and 373 patients in the RT group. Thirty-eight patients being operated on at other hospitals, 2 patients undergoing neoadjuvant ADT, and 6 patients with adjuvant RT after operation in the RP group were excluded. We restricted patients to those with RT and 1.5–3 years ADT on the basis of NCCN guideline recommendations. A total of 166 patients were thus excluded. Three patients who experienced biochemical recurrence (BCR) during ADT treatment were also excluded. Nine patients lost to follow-up, with 3 in the RP group and 6 in the RT group, were also excluded. Of these, 309 patients were included in our study: 111 in the RP group and 198 in the RT group.

### Statistical methods for clinical variables and definition of outcomes

A review was conducted of retrospectively obtained clinical data taken from the electronic medical records. Patient information was anonymized and de-identified prior to analysis. For each group, descriptive statistics were used to summarize the clinical presentation (age at diagnosis, biopsy GS grade group, PSA at diagnosis (iPSA) and clinical T stage by MRI). Continuous variables were shown as median (range) and categorical variables as number (percentage). The Mann–Whitney U-test was performed to determine statistical significance for continuous variables between the groups while chi-square test or Fisher’s exact test was used for categorical variables. Propensity score (PS) matching analysis was performed to reduce the selection bias in this observational study, achieving a more comparison between the two groups. The PS was calculated using a logistic regression model and covariates entered into the PS matching model were as follow: age at diagnosis, biopsy GS grade group, iPSA and clinical T stage. PS matching was performed using a 1:1 matching method. The macro language made best matches first and next-best matches next. The PS matching sample was under SAS 8.2 Knowledge of logistic regression analysis. Of these, 136 patients were included in our study. BCR was defined as two consecutive times of PSA ≥ 0.2 ng/ml in the RP group and rising PSA of 2 ng/ml above the nadir (Phoenix criteria) in the RT group. Local recurrence was defined as lymphadenopathy or tumor recurrence in the pelvis by computed tomography (CT) or MRI. Metastasis was defined as distant metastasis in imaging (CT, MRI or bone scan). Kaplan–Meier analysis was performed to analyze BCR-free survival, local recurrence-free survival, metastasis-free survival and overall survival. To avoid immortal-time bias, the elapsed time for BCR-free survival analysis was calculated from the end of ADT treatment in the RT group and the operation date in the RP group to the date of BCR (Supplementary Figure). In local recurrence-free survival, we calculated the follow-up time from the end of RT treatment. Univariate and multivariate Cox proportional hazards models were used to analyze the relationships between clinical variables and oncologic outcomes including BCR, local recurrence, metastasis and overall survival. All statistical analyses were performed using SPSS version 22.0 (IBM, Armonk, USA). Two-sided *p* values were calculated and a level of < 0.05 was considered statistically significant.

### Ethical considerations

The study was approved by the Institutional Review Board and Ethics Committee of National Taiwan University Hospital (IRB 201911084RINC) and all methods were performed in accordance with relevant guidelines and regulations. Informed consent from all subjects could be eliminated in this retrospective study according to Institutional Review Board and Ethics Committee of National Taiwan University Hospital (IRB 201911084RINC) regulation.

## Results

Patient characteristics are summarized in Table 1. A total of 309 patients were included. There were 111 patients (35.9%) in the RP group and 198 patients (64.1%) in the RT group. The median follow-up period was 62.8 months (interquartile range, IQR 33.8–89.5) in the RP group and 56.1 months (IQR 41.4–85.9) in the RT group (*p* = 0.542). RT patients were older than RP patients (*p* < 0.001). A total of 171 patients (86.4%) in the RT group were older than 65 years, compared to 51 (45.9%) in the RP group. RT patients had higher iPSA levels (*p* < 0.001) and GS grade group (*p* < 0.001). There were 91 patients (46.0%) in the RT group with iPSA levels ≥ 20 ng/ml and 21 patients (18.9%) in the RP group. A total of 138 patients (69.6%) in the RT group had GS grade group ≥ 3, compared to 56 (48.6%) in the RP group. RT patients also had more advanced clinical T stage (*p* < 0.001). In the RT group, 90 patients (45.4%) had T stage ≥ T3b, and 7 patients (3.5%) had T stage 4. In the RP group, 19 patients (17.1%) had T stage ≥ T3b and no patient had T stage 4. In the RP group, 55 (49.6%) clinical T3 diseases defined by MRI were downgraded to pathologic T2.

**Table 1:** Baseline characteristics of patients.

	Full cohort (N = 309)			Propensity score-matched patients (N = 136)		
	RP	RT with ADT	*p* value	RP	RT with ADT	*p* value
Number	111 (35.9%)	198 (64.1%)		68 (50%)	68 (50%)	
Follow-up (months)	62.8 (33.8–89.5)	56.1 (41.4–85.9)	0.542	54.2 (33.6–84.4)	62.5 (43.2–89.6)	0.071
**Age**						
< 65	60 (54.1%)	27 (13.6%)	< 0.001	21 (30.9%)	16 (23.5%)	0.335
≥ 65	51 (45.9%)	171 (86.4%)		47 (69.1%)	52 (76.5%)	
**PSA at diagnosis**						
< 10	50 (45.0%)	44 (22.2%)	< 0.001	22 (32.4%)	24 (35.3%)	0.932
10–20	40 (36.0%)	63 (31.8%)		32 (47.1%)	31 (45.6%)	
≥ 20	21 (18.9%)	91 (46.0%)		14 (20.6%)	13 (19.1%)	
**Gleason score grade group**						
< 3	57 (51.4%)	60 (30.3%)	< 0.001	23 (33.8%)	27 (39.7%)	0.477
≥ 3	54 (48.6%)	138 (69.7%)		45 (66.2%)	41 (60.3%)	
**MRI T-stage**						
3a	92 (82.9%)	108 (54.5%)	< 0.001	53 (77.9%)	57 (83.8%)	0.383
3b	19 (17.1%)	83 (41.9%)		15 (22.1%)	11 (16.2%)	
4	0 (0%)	7 (3.5%)		0 (0%)	0 (0%)	

The Mann–Whitney U-test was performed to determine statistical significance for continuous variables between the RP and the RT group while chi-square test or Fisher’s exact test was used for categorical variables. To reduce selection bias, we performed propensity score (PS) matching at a 1:1 ratio of the following variables: age at diagnosis, biopsy GS grade group, PSA at diagnosis and clinical T stage. Two-sided *p* values were calculated and a level of < 0.05 was considered statistically significant.

*RP* radical prostatectomy, *RT* radiotherapy, *ADT* androgen deprivation therapy.

After propensity score (PS)-matched, there were 68 patients in both groups. Patient characteristics were well balanced and are summarized in Table 1. The median follow-up period was 54.2 months (IQR 33.6–84.4) in the RP group and 62.5 months (IQR 43.2–89.6) in the RT group (*p* = 0.071). There was no difference in age (*p* = 0.335), iPSA level (*p* = 0.932), GS grade group (*p* = 0.941) or T stage (*p* = 0.383) between the two groups.

**Figure 1:**
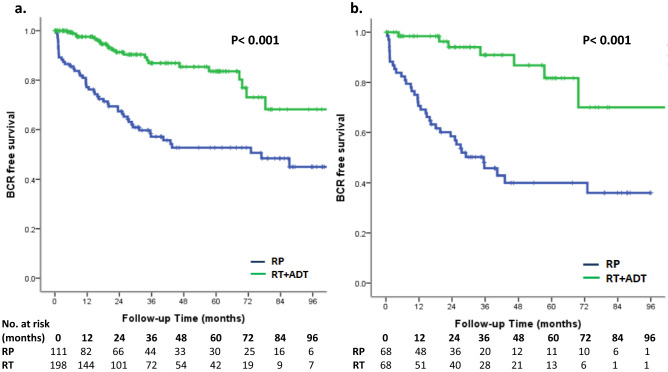
Kaplan–Meier analysis of biochemical recurrence free survival in (**a**) all cohort patients and (**b**) propensity-score matched patients. For each treatment group, we generated Kaplan–Meier survival curves for biochemical recurrence (BCR) according to management method, and calculated a log-rank test to evaluate the association of BCR with different management method. We also compared the BCR free survival between all cohort patients and propensity-score matched patients.

**Figure 2:**
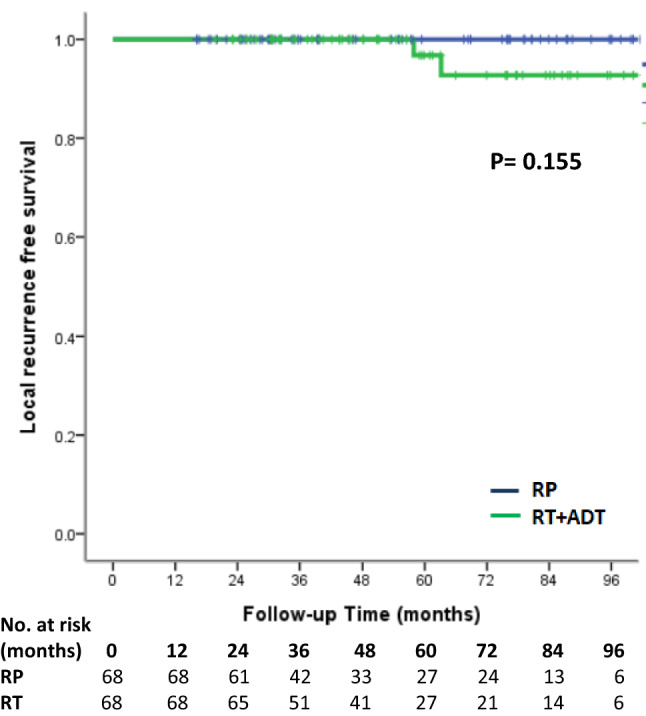
Kaplan–Meier analysis of local recurrence free survival in propensity-score matched patients. For each treatment group, we generated Kaplan–Meier survival curves for local recurrence according to management method, and we also calculated a log-rank test to evaluate the association of local recurrence with different management method among propensity-score matched patients.

**Figure 3:**
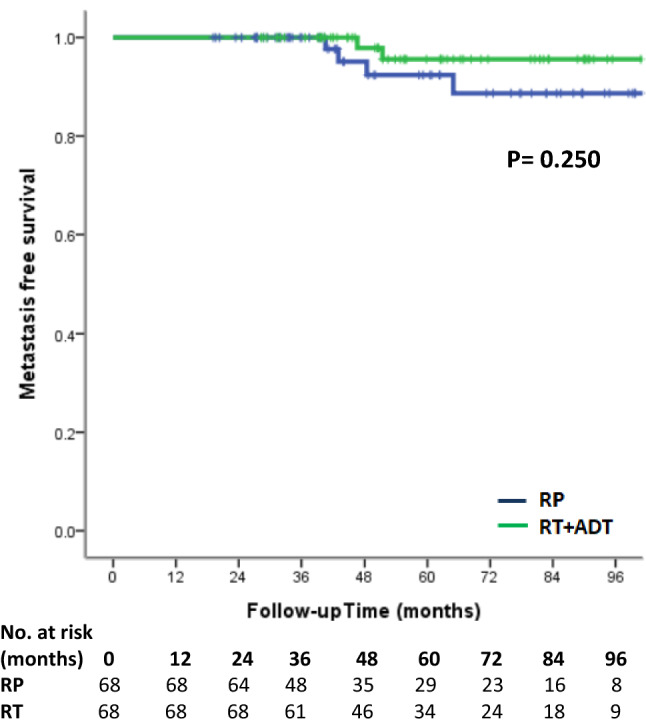
Kaplan–Meier analysis of metastasis free survival in propensity-score matched patients. For each treatment group, we generated Kaplan–Meier survival curves for metastasis according to management method, and we also calculated a log-rank test to evaluate the association of metastasis with different management method among propensity-score matched patients.

**Figure 4:**
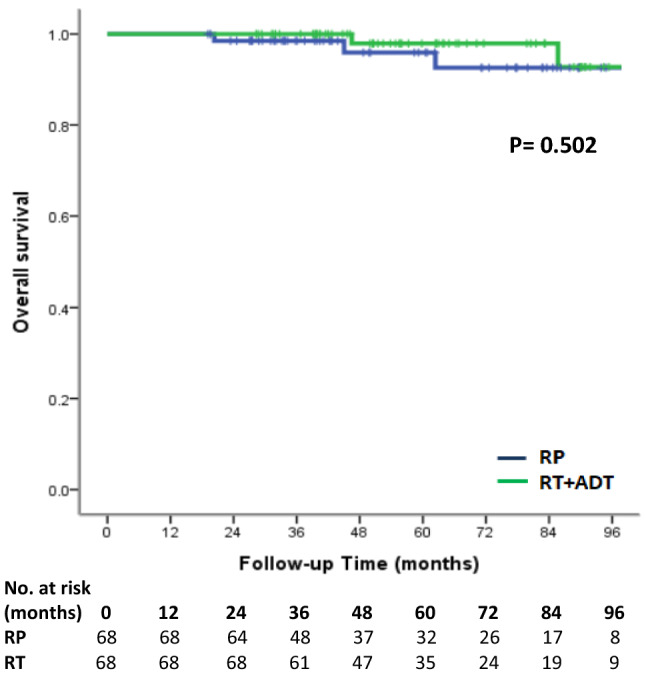
Kaplan–Meier analysis of overall survival in propensity-score matched patients. For each treatment group, we generated Kaplan–Meier survival curves for overall survival according to management method, and we also calculated a log-rank test to evaluate the association of overall survival with different management method among propensity-score matched patients.

Treatment with RP had a higher risk of biochemical recurrence compared to the RT group. (log-rank test, *p* < 0.001, Fig. 1). The univariate and multivariate analyses of predictors of BCR are demonstrated in Table 2. PS-matched patients with RT treatment were associated with reduced risk of BCR (hazard ratio (HR) 0.16, 95% confidence interval (95% CI) 0.07–0.37, *p* < 0.001). There was a higher risk of BCR in patients with more advanced MRI T stage (*p* = 0.014). GS grade group could predict BCR only in univariate analysis (*p* = 0.019) but failed to predict BCR in multivariate analysis (*p* = 0.065). In Kaplan–Meier analysis, there was no significant difference in local recurrence-free survival (log-rank test*, p* = 0.155, Fig. 2), metastasis-free survival (log-rank test*, p* = 0.250, Fig. 3) and overall survival (log-rank test, *p* = 0.502, Fig. 4) between the two groups. In Table 3, there were no independent variables, including treatment methods, to predict local recurrence-free survival, metastasis-free survival and overall survival in the univariate analysis.

**Table 2:** Cox regression for biochemical-recurrence free survival in propensity score-matched patients.

Univariate			Multivariate		
Group		*p* value	Group		*p* value
RP	Ref		RP	Ref	
RT	0.17 (0.08–0.38)	< 0.001	RT	0.16 (0.07–0.37)	< 0.001
**Age**					
< 65	Ref	0.224			
≥ 65	0.68 (0.37–1.27)				
**PSA at diagnosis**					
< 10	Ref	0.207			
10–20	1.85 (0.93–3.69)				
≥ 20	1.37 (0.56–3.34)				
**Gleason score grade group**			**Gleason score grade group**		
< 3	Ref	0.019	< 3	Ref	0.065
≥ 3	2.21 (1.14–4.30)		≥ 3	1.89 (0.96–3.73)	
**MRI T-stage**			**MRI T-stage**		
3a	Ref	0.010	3a	Ref	0.014
3b/4	2.36 (1.23–4.52)		3b/4	2.34 (1.19–4.60)	

Cox proportional hazards models with 95% confidence interval were used to analyze the relationships between clinical variables and biochemical recurrence. We calculated multivariate cox regression analysis when clinical variables with *p* < 0.2 at univariate cox regression analysis. Two-sided *p* values were calculated and a level of < 0.05 was considered statistically significant.

*RP* radical prostatectomy, *RT* radiotherapy.

**Table 3:** Cox regression univariate analysis for local-recurrence free survival, metastasis free survival and overall survival in propensity score-matched patients.

Group	Local-recurrence free survival		Metastasis free survival		Overall survival	
		*p* value		*p* value		*p* value
RP	Ref	0.470	Ref	0.268	Ref	0.509
RT	66.3 (0.00–5,813,788)		0.38 (0.07–2.09)		0.55 (0.09–3.3)	
**Age**						
< 65	Ref	0.576	Ref	0.536	Ref	0.536
≥ 65	36.5 (0.00–10,789,905)		1.9 (0.23–16.9)		1.9 (0.23–16.9)	
**PSA at diagnosis**						
< 10	Ref	0.985	Ref	0.857	Ref	0.951
10–20	0.78 (0.05–12.5)		1.6 (0.29–8.8)		0.74 (0.11–5.3)	
≥ 20	0.00 (0.00–)		0.00 (0.00–)		0.99 (0.09–10.9)	
**Gleason score grade group**						
< 3	0.02 (0.00–1504)	0.483	0.02 (0.00–15.70)	0.249	5.32 (0.59–47.97)	0.136
≥ 3	Ref		Ref		Ref	
**MRI T-stage**						
3a	Ref	0.300	Ref	0.363	Ref	0.923
3b/4	4.4 (0.27–70.8)		2.2 (0.40–12.0)		1.12 (0.12–10.1)	

Cox proportional hazards models with 95% confidence interval were used to analyze the relationships between clinical variables and oncologic outcomes including local recurrence, metastasis and overall survival. Two-sided *p* values were calculated and a level of < 0.05 was considered statistically significant.

*RP* radical prostatectomy, *RT* radiotherapy.

## Discussion

To date, no published randomized trial has so far compared RP to RT plus ADT in locally advanced PC^11^. The most optimal strategy in this high-risk subpopulation remains controversial. Most retrospective studies have inevitable selection bias, heterogeneous treatment protocols and unclear outcome definitions^10^. In this retrospective study, used the PS-matched method, strictly limited patient enrollment, and clear-cut outcome definition, trying to provide direction for decision-making. We found that treatment with RP had a higher risk of BCR compared to the RT group among locally advanced PC patients. Furthermore, there was no significant difference in local recurrence, metastasis or overall survival. Different treatment-related complications in each strategy should not be overlooked during patient counseling. The current results demonstrated valuable clinical information that may impact the strategy for disease management.

The reason to choose MRI as a reference of the clinical T stage is to better illustrate extracapsular invasion and for comparability between surgery and radiotherapy groups. Conventionally, DRE has been the fundamental method to detect PC and is used as a clinical T stage reference. However, DRE is a subjective test and could not detect anteriorly located tumors. That is why multiple risk stratification models, combining PSA, GS and DRE, have been developed to better evaluate the risk of PC. In addition, DRE often overestimates tumor staging and may not evaluate locally-advanced disease accurately^12^. In the last decade, MRI of the prostate has become a vital diagnostic tool for local staging, while PI-RADS is generally applied for global standardization. One meta-analysis, including 9,796 patients, reviewed studies that used MRI for detection of extracapsular extension (ECE), seminal vesicle invasion (SVI) or overall stage T3 PC^7^. The pooled data for ECE, SVI and overall stage T3 detection showed corresponding sensitivity and specificity of 0.57 (95% CI 0.49–0.64) and 0.91 (95% CI 0.88–0.93), 0.58 (95% CI 0.47–0.68) and 0.96 (95% CI 0.95–0.97) and 0.61 (95% CI 0.54–0.67) and 0.88 (95% CI 0.85–0.91), respectively. Because of microscopic involvement confirmed by resected prostate specimen regardless of size, high specificity and low sensitivity of MRI were found on meta-analysis. To make comprehensive decision-making regarding preservation of neurovascular bundles, high resolution of pre-operative images with good sensitivity is crucial. Draulans et al. revealed that the use of MRI images instead of DRE upstaged clinical T stage (33%) and European Association of Urology (EAU) risk grouping (31%)^13^. MRI showed a higher sensitivity than did DRE for detection of non-organ-confined PC (59 vs 41%, *p* < 0.01) in terms of corresponding pathologic T stage, and furthermore, with incorporation of MRI instead of DRE staging alone, the surgical treatment strategy would be altered in 27% of patients. In other words, DRE or transrectal ultrasonography (TRUS) alone is not accurate enough for local staging (T stage), and MRI is still one of the best imaging tools for assessing ECE in clinical practice^14^.

Historically, men with locally advanced PC have been managed mostly with RT with ADT, while RP has been discouraged due to concerns about positive surgical margin, inadequate local control and side effects^15^. Roach et al. noted that RT combined with ADT has lower cancer-specific mortality (CSM), distant metastasis and biochemical failure, without increasing the risk of fatal cardiac effect in locally advanced PC^16^. However, the use of RP has increased gradually and the proportion of patients treated with RP or RT has changed over time. In the Surveillance, Epidemiology and End Results (SEER) database (2004–2014), Marco et al. identified 5500 cT3N0-1M0 PC patients and noted that CSM was significantly lower after RP compared to RT^17^. Another Swedish observational study, including 34,515 patients, showed that RP has better survival than RT. Younger men and those with intermediate- or high-risk localized PC benefit more from surgery during 15 years of follow-up^18^. Consistently, a higher PSA and older age were noted in the RT group in the current cohort. In summary, the pathologic reports of prostatectomy, the dose of RT and duration of ADT treatment were not described in the majority of studies, and the discrepancies might influence the CSM^17,18^. One ongoing prospective randomized SPCG-15 trial with a similar trial setting as the current study might deliver valuable information regarding this specific population in the future^11^.

Hackman et al. noted that adjuvant RT following prostatectomy prolonged biochemical recurrence-free survival compared with RP alone^19^. However, there was no difference in local recurrence, overall survival or cancer-specific survival. On the other hand, more adverse events were noted in the adjuvant group, where 56% experienced grade 3 adverse events versus 40% in the observational group (*p* = 0.016). Adjuvant RT caused more toxicity and could impair quality of life. Because of the above reasons, all patients receiving RP in our cohort were observed following prostatectomy, which enabled us to compare the effect of surgery alone without adjuvant RT confounding. In our cohort, patients in the RP group had a higher risk of BCR compared to the RT group. This might be explained by the lack of adjuvant therapy in the RP group and the dose of RT. Mitchell et al. found that 59% of patients suffered from BCR (defined as a PSA level > 0.4 ng/ml) after RP for cT3 disease but that only 12.9% of patients received adjuvant RT in their study^20^. Aligned with the Mitchell et al. study, the current study also showed that 45% of patients suffered from BCR in the RP group. When trying to compare the therapeutic effect between RP and RT, the additional peri-operative RT in the RP group led to some bias, so the current study only included patients with RP alone.

Comparing to RT plus LTADT, one of the irreplaceable benefits of RP for patients in locally advanced PC is the ability to acquire accurate pathologic staging. Pathologic staging provided more reliable information to guide adjunctive therapies based on more precise data than bio-clinical variables including clinical T stage, biopsy GS or PSA. Indeed, 22 to 63% of PC initially defined as high risk have been found to have organ-confined disease following RP^21^. Stephen et al. also showed that 57% of patients initially classified as D’Amico high-risk PC have organ-confined disease at RP^22^. In addition, discrepancies in GS have frequently been found between biopsy and RP, such as up to 52.2% of GS over 8 tumors at biopsy had score downgrading at RP^23^. In the Mayo Clinic, 26% of PC patients with clinical T3 were downgraded to pathologic T2^20^. Similarly, the cT3–4 stage has shown to be inaccurate in up to 33% of cases at RP^24^. In our cohort, 55 (49.6%) clinical T3 diseases defined by MRI were downgraded to pathologic T2 at RP. In addition, 50 out of 60 (83%) and 42 out of 51 (82%) patients before and after 2015 respectively were found to have clinical T3a disease on MRI, and there was no statistically different prevalence (*p* = 0.891). Among these patients, 33 patients (55%) diagnosed before 2015 were downgraded to pathologic T2, compared to 22 patients (43.1%) diagnosed after 2015; and despite a trend toward decreased discrepancy, there was no statistical difference (*p* = 0.076). Reviewing the accuracy of MRI imaging for local staging of PC, MRI images demonstrated moderate sensitivity for clinical T3 following prostatectomy (area under the curve of ROC: 0.61, 95% CI 0.54–0.67)^7^. The current meta-analysis shows that MRI has high specificity but low sensitivity^7^. Conventionally, radiologists have focused on high-specificity reading to minimize unnecessary exclusion of men from curative treatment. In addition, MRI is limited for detection of focal ECE, which might increase its discrepancy^25^.

Local disease control using different doses of RT in patients with PC is a critical issue. The total dose of 6000–7020 centigray (cGy) is currently recommended for localized advanced PC on the basis of the most updated guideline^8^. In our study, nearly all patients received RT with 7800 cGY in 39 fractions. Nevertheless, the most ideal total dose of RT is still under investigation and could influence oncologic outcomes. Local failure after RT is an independent factor of overall survival, CSM and metastasis-free survival in high-grade localized PC^26^. Few randomized control trials (RCTs) have revealed that dose escalation (range 7400–8000 cGy) has a significant impact on BCR, metastasis and CSM^27–29^. There are still inconsistent data on the effect of oncologic outcomes. However, the MRC RT01 RCT demonstrated that dose escalation (7400 vs 6400 cGy) showed an advantage in BCR, but the advantage did not translate into the improvement of overall survival^30^. Francolini et al. noted that dose-escalated pelvic radiotherapy and boost on positive lymph nodes were effective approaches to improve BCR^31^. Locally advanced PC or lymph node positive disease might benefit from dose-escalation. Some uncertainty still exists regarding different subpopulations. A retrospective analysis of the US National Cancer Database, including 42,481 patients receiving RT, showed that dose escalation is associated with improved overall survival in patients with intermediate- or high-risk PC, but not with low-risk PC^32^.

Dose escalation might be related to more toxic effects. Michalski et al. noted that dose escalation (7920 vs 7020 cGy) showed higher rates of toxic effects^29^. The 5-year rates of 2 or greater rectum and genitourinary tract toxic effects were 21 and 12% in high-dose arm and 15 and 7% with 7020 cGy. When dose escalation was applied, the rates of severe late side effects (> grade 3) were 2–3% for rectum and 2–5% for the genitourinary tract^33^. Consistently, 7 patients (3.5%) suffered from severe radiation cystitis and received blood clot evacuation in our RT group. In terms of treatment-related complications, 25 cases of complication were recorded in the RP group with the majority no more than Clavien–Dindo Grade III, while 8 major complications were recorded in the RT group. Among the 25 complications in the RP group, 12 were Clavien–Dindo Grade I, 8 were Grade II and 5 were Grade III (3 patients, lymphatic leakage; 1 patient, pleural effusion; 1 patient, need for laparoscopic foreign body removal for incarcerated drainage tip). In the RT group, 196 patients (98.99%) received high-dose RT with 7800 cGY in 39 fractions. Among the 8 major complications, 1 patient died because of refractory radiation proctitis bleeding, while 7 patients suffered from severe radiation cystitis and needed blood clot evacuation. In addition, among the 8 major complications, 6 patients received volumetric modulated arc therapy (VMAT) and 2 patients received intensity-modulated radiation therapy (IMRT). Furthermore, IMRT, a major step with a wider therapeutic index, could be obtained from refined assessment of radiation-induced morbidity at an individual level^34^. In summary, although high-dose RT with LTADT showed better biochemical control, the mid-term survival outcomes were similar to those in treatment with RP in our cohort, and the relatively higher complications rate should not be underestimated. The optimal treatment strategies still need a large cohort to determine the risk–benefit.

To the best of our knowledge, this is the first study to compare RP alone, without adjuvant RT, with RT plus LTADT and take the immortal-time bias into consideration. RP alone allowed us the opportunity to observe the natural course of disease after prostatectomy. The potential for immortal time bias, also known as guarantee-time bias, exists whenever an analysis that is timed from enrollment is compared across groups defined by a classifying event occurring sometime during follow-up^35^. It could be challenging for investigators to recognize when immortal time bias influences the outcome of analyses. When treating BCR as the outcome, the PSA level would be influenced by ADT treatment and interfered with the evaluation of outcome if immortal time bias was not well considered. However, there were scarce data exploring this bias in previous studies, and investigations retrieved from a database showed heterogeneous ADT duration^36^. In our cohort, patients in the RT group received pre-defined and consistent duration of 1.5–3 years of ADT. If we were to extend the duration of ADT treatment, the RT group would have longer BCR-free survival, which would influence the outcome. As a result, the distinct feature of the current study is that we calculated BCR time from the end of ADT treatment to avoid immortal-time bias.

Nevertheless, there were some limitations in our study. First, the retrospective, short follow-up period and small sample size limited extensive analysis and we could only analyze mid-term overall survival within 5 years. There were only 10 deaths (3.2%) in our cohort and no cancer-related death event for analysis with enough statistical power. Second, patient distribution was unbalanced in the two groups. Patients in the RT group were older and had higher iPSA, higher GS and more advanced T stage compared to patients in the RP group. However, the effect could be minimal after adjusting by PS-matched and multivariate analysis. Third, the matched sample size was less than 50% and the incomplete matching might influence our results. However, decreasing the sample size from 1000 to 40 did not alter Type I error rate and led to relative biases below 10% unless the true confounders related only to the outcome are not included in the PS model^37^. Fourth, there were no records of Charlson Comorbidity Index and ECOG in our study. These factors might have led to unavoidable selection bias, influencing the choice of treatment methods, and somehow impacting survival. Fifth, erectile function and continence were closely related to the outcome of satisfaction. In our cohort, we only explored the oncological outcomes, and not the functional outcomes. Sixth, whether the clinical benefit in BCR control remains when compared to patients receiving RP plus adjuvant RT is beyond the scope of the current study. Finally, we used MRI T stage because clinical T stage by DRE had inherent bias due to its subjective nature and potentially caused variability. However, there was a lack of central review of the MRI images. After analyzing the discrepancy rate of MRI reports between different time periods, it remained stable with no statistical difference. We therefore believed that the inter-observer variation in the current study was limited because all the radiologists in this high-volume tertiary referral medical center were well experienced.

## Conclusion

Among patients with locally advanced PC, treatment with RP had a higher risk of BCR but no significant difference in the risk of local recurrence, metastasis, and overall survival compared to RT plus LTADT therapy. There is insightful information supporting a clinical benefit in BCR control for patients undergoing RT plus LTADT compared to RP. A further prospective and long follow-up period study for patients with advanced PC would be necessary.

## Supplementary Information

Supplementary Figure S1.
