# Supplementary material for: Propensity score matching analysis comparing radical prostatectomy and radiotherapy with androgen deprivation therapy in locally advanced prostate cancer
Source: Sci Rep. 2022 Jul 21;12:12480. doi: 10.1038/s41598-022-16700-7 (PMC9304348; doi:10.1038/s41598-022-16700-7)

**Supplementary Figure. Illustration of the definition of biochemical recurrence (BCR)-free survival (A) and follow-up period (B).**

To avoid immortal time bias, the elapsed time for BCR-free survival analysis was calculated from the end of androgen deprivation therapy treatment in the radiotherapy (RT) group (Upper bar) and the operation date in the radical prostatectomy (RP) group to the date of BCR (Lower bar); The follow-up period was calculated from the date of diagnosis in both RT and RP groups, respectively.


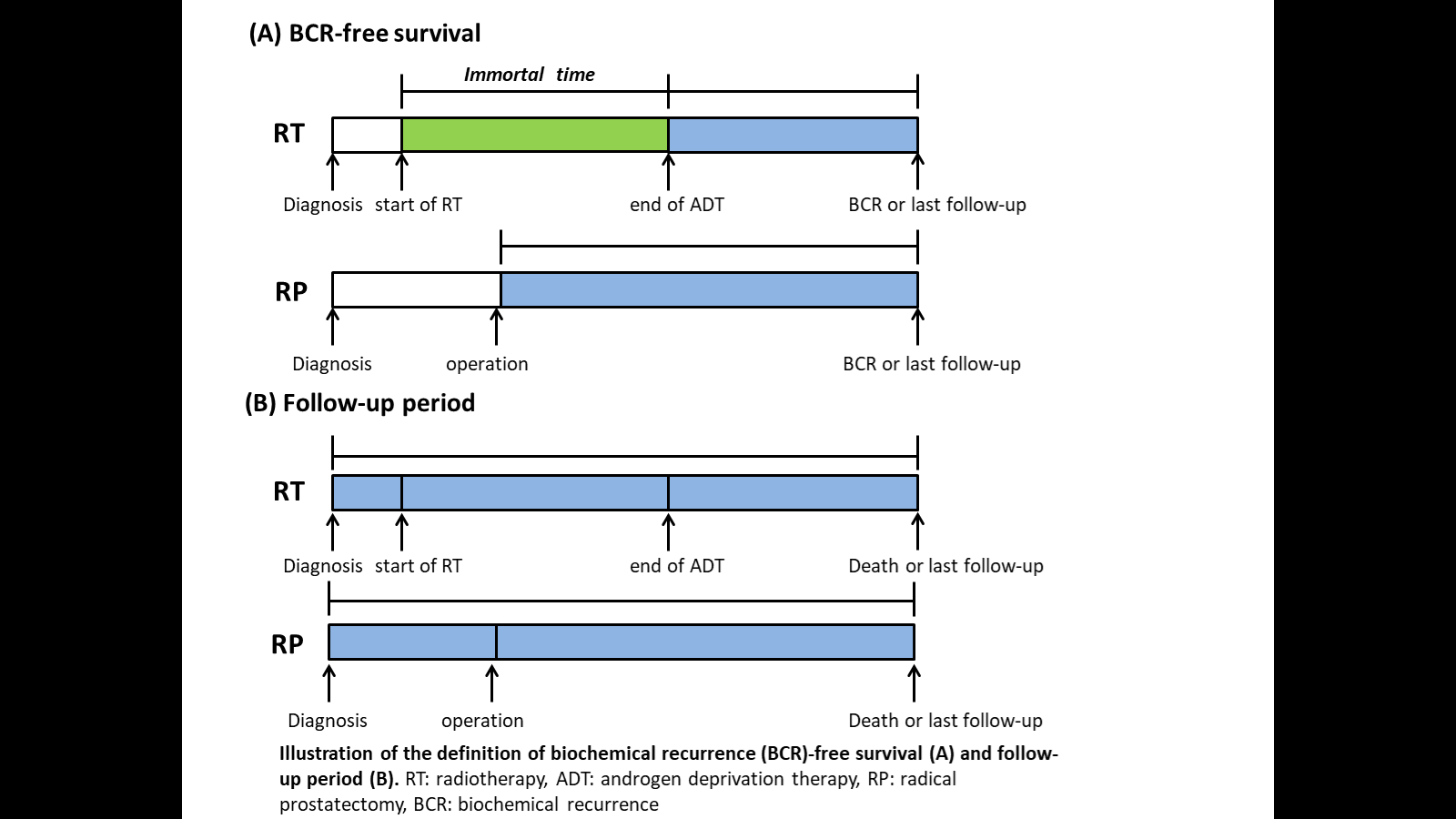

Supplement: Supplementary file 1 — Supplementary Figure S1. [file 41598_2022_16700_MOESM1_ESM.docx]
